# Acoustic analysis reveals a new cryptic bush–cricket in the Carpathian Mountains (Orthoptera, Phaneropteridae)

**DOI:** 10.3897/zookeys.254.3892

**Published:** 2012-12-21

**Authors:** Ionuţ Ştefan Iorgu

**Affiliations:** 1 “Grigore Antipa” National Museum of Natural History, Kiseleff blvd. 1, 011341, Bucharest, Romania

**Keywords:** *Isophya*, bioacoustics, taxonomy, Carpathians

## Abstract

A new morphologically cryptic species of phaneropterid bush–cricket from the genus *Isophya* is described from the Eastern Carpathian Mountains: *Isophya dochia* **sp. n.** Sound analysis and morphological details are discussed in the paper comparing the new species with several *Isophya* species having similar morphology and acoustic behavior.

## Introduction

One of the largest and most enigmatic phaneropterid genera, with 90 species known and 45 species present in Europe, *Isophya* Brunner von Wattenwyl inhabits Southern and Eastern Europe, Asia Minor and Caucasus up to Kazakhstan, Iran and Iraq (Eades et al. 2012).

Dissimilar to morphological homogeneity in genus *Isophya*, the specific structure of male acoustic signals shows clear differences in close related species and is used as the most effective tool for identifying and clarifying taxonomic relations (Heller et al. 2004, Chobanov 2009, Orci et al. 2010a, 2010b, Szövényi et al. 2012). Contrary to this general opinion, a recent study on several related *Isophya* species from Asia Minor suggests that, in some cases, evolutionary changes in song appear slower than in morphology (Sevgili et al. 2012). In this genus, the male–female pair is usually formed during an acoustic duet; females locate the males by phonotaxis and respond to their songs with simple impulses (Orci et al. 2001, Orci 2007, Orci et al. 2010a, 2010b) or complicated click–series (Iorgu 2012) within a species–specific time span.

The *Isophya* species are very interesting subjects to study: apart from their remarkable behavior, most of the species have restricted distribution areas and present a large number of endemics. For the time being, 16 *Isophya* species are known to occur in the Carpathian Mountains (Nagy 2005, Heller 2012). Recently, three new species have been described from the Eastern Carpathian Mountains: *Isophya sicula* Orci, Szövényi and Nagy, 2010; *Isophya ciucasi* Iorgu and Iorgu, 2010 and *Isophya nagyi* Szövényi, Puskás and Orci, 2012.

In the summer of 2005, several bush–crickets were collected from the area called “Poliţa cu Ariniş”, close to the subalpine meadows of Ceahlău Mountains. Studying only the morphological characters, they were identified back then as *Isophya camptoxypha* (Fieber) and up to the summer of 2010 no acoustic data of this geographically isolated population were available. With the first recorded songs, its status had to be changed in a new taxa, perfectly morphologically cryptic: *Isophya dochia* sp. n.

## Material and methods

Audio recordings were taken with an Edirol R–09HR digital recorder (microphone frequency response 20–40000 Hz, sampling rate of 96000 Hz, 24 bit amplitude resolution). In the field, we used an Edirol CS-15R unidirectional external microphone attached to the digital recorder (frequency response 200–17000 Hz). Temporal and spectral sound analyses were performed with the software Audacity 2.0.2.

**Figures 1–5.**
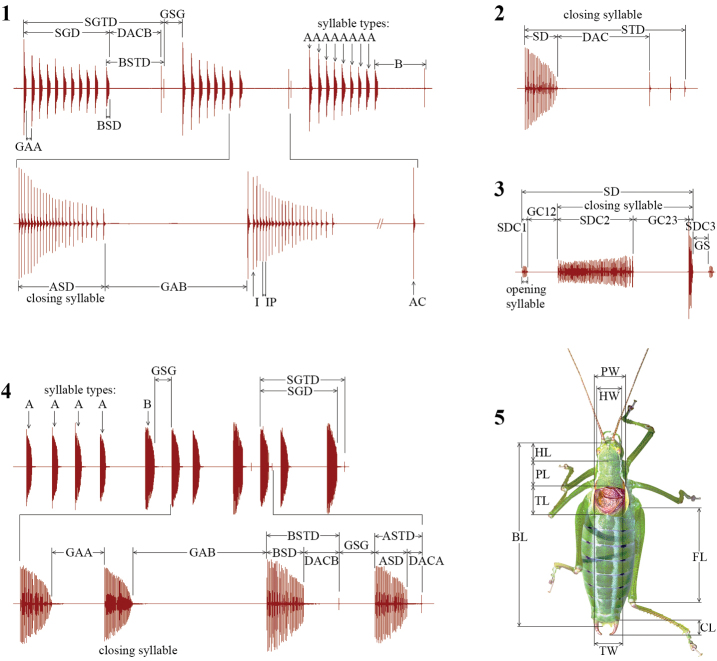
Schematics of studied song (**1** *Isophya dochia* sp. n. **2** *Isophya camptoxypha* **3** *Isophya nagyi* **4** *Isophya harzi*) and morphological (**5** *Isophya dochia* sp. n.) characters.

Song terminology and abbreviations are adapted from Heller et al. 2004, Orci et al. 2005 and Orci et al. 2010a (Figs 1–4; see Appendix 1: Isophya song abbreviations).

Morphological traits were examined with a stereomicroscope and the following characters were measured for 20 males and 20 females of the new species: body length (BL), head width (HW), head length (HL), pronotum maximum width (PW), pronotum length (PL), left tegmen maximum width (TW), tegmina length (TL), cercus length (CL) and femur length (FL) (Fig. 5).

Photos were taken with Canon EOS 600D DSLR camera and Canon 100 mm 1:1 and Canon MP–E 65 mm 5:1 macro lenses, using photo stacking method for morphological characters. Movements of tegmina during sound production have been video recorded with the same camera, with the external microphone attached.

The distribution area map was drawn using the altitude layer from Jarvis et al. (2008).

Type specimens are preserved in the collections of “Grigore Antipa” National Museum of Natural History, Bucharest, Romania.

## Data resources

The data underpinning the analyses reported in this paper are deposited in the Dryad Data Repository at doi: 10.5061/dryad.256qh

## Taxonomy

### Isophya dochia sp. n.

urn:lsid:zoobank.org:act:E527F61F-59EC-4761-8E23-495306978BB6

http://species-id.net/wiki/Isophya_dochia

Figs 1 6 [Fig F3] [Fig F4] –26 32 38 44 45, 51, 57, 63, 69 75

#### Type locality.

Romania, Eastern Carpathian Mountains, Ceahlău Mountains.

#### Type material.

Holotype: male. Original label: “România, Munţii Ceahlău, Poliţa cu Ariniş, 46°57.90'N, 25°56.32'E, alt. 1620 m, 09.08.2005, Leg. Iorgu I. Ş.”

Paratypes: 10 ♂♂ 7 ♀♀, labeled: “România, Munţii Ceahlău, Cabana Dochia, 46°57.94'N, 25°57.02'E, alt. 1740 m, 02.07.2010, Leg. Iorgu I. Ş.”; 6 ♂♂ 5 ♀♀, labeled: “România, Munţii Ceahlău, Piatra Lată, 46°57.73'N, 25°56.93'E, alt. 1720 m, 10.07.2012, Leg. Iorgu I. Ş”, a microSD card containing audio and video records of male song attached; coll. “Grigore Antipa” National Museum of Natural History, Bucharest, Romania.

#### Audio recordings.

8 ♂♂, 5 July 2010, in laboratory, air temperature 25°C; 17 ♂♂, 10 July 2012, in the field, air temperature 20°C; 5 ♂♂ 5 ♀♀, 12 July 2012, in laboratory, air temperature 24°C (see Appendix 2: Isophya dochia sp n audio and Appendix 3: Isophya dochia sp n video).

#### Comparative material

(see Appendix: 4 Isophya comparative material).

#### Description

(Figs 12–22; Table 1). Male. Fastigium verticis slightly tapering frontward, half as wide as scapus, with a dorsal groove (Fig. 12). Head length 1.7 times the pronotum length and head width about 1.3 times the maximum pronotum width. Pronotum saddle shaped from a lateral view, paranota with concave dorsal margins, anterior and ventral borders straight, posterior edge moderately convex. Pronotal disc slightly constricted in the transverse sulcus area, with lateral carinae marginally divergent in mesozona and convex in metazona (Figs 12, 13). Wings as long as or slightly longer than pronotum, with reticulate venation, usually surpass the posterior edge of first abdominal tergite. Cu2 vein length about 3/4 the posterior margin of pronotum; angle between cubital veins about 70°. Speculum large and rectangular. Edge of tegmen at distal end of Cu2 vein forms an obtuse angle of about 130° (Fig. 12). During the quiet wing openings in song production, the white anterior border of tegmen is well visible, but its role has not been established yet (see Appendix 3: Isophya dochia sp n video). Stridulatory file arcuate, 2.2–2.4 mm long, counts 82–89 teeth; distal teeth larger and rarer than proximal ones (Fig. 16). Epiproct about twice as wide as long; cercus slender, narrowing towards tip, slightly curved in its apical fourth, with fine, small hairs; terminal denticle located in middle of cercus apex (Fig. 14). Subgenital plate elongated, narrowed apically, with triangular apical incision (Fig. 15). Hind femur about 4–4.3 times the pronotum length, without ventral spines. Coloration green, densely punctuated with fine, dark green and brown spots. Several males with two dorso–lateral, parallel stripes from pronotum to end of abdomen, red, orange, white, violet or yellow colored. Antennae greenish–brown or reddish–brown, with light brown or green scapus. Compound eyes bicolor: upper part brownish–red and lower part green. A yellowish or white band begins behind the eye and ends at posterolateral angle of wing. Tegmina brown, dark brown or dark red, apically green and costal margin greenish–white or yellowish–white. Cerci brown or reddish–brown, green at base. Ventral side of body yellowish–green. Femora, tibiae and tarsi usually green, brownish or reddish.

Female. Fastigium roughly as in male (Fig. 17). Head length 1.7 times the pronotum length and head width about 1.3 times the maximum pronotum width. Pronotum disc marginally enlarged in its posterior part, with straight lateral carinae, paranota as in males (Figs 17, 18). Wing with dense reticulate venation, surpass the posterior margin of first abdominal tergite. Stridulatory bristles located on cubital veins in the inner latero–posterior part of right tegmen (Fig. 19). Cercus short, hairy, conical (Fig. 20). Subgenital plate rounded, narrow, about twice as wide as long (Fig. 21). Ovipositor short, upcurved, 1.9–2.1 times the pronotum length, upper margin with 9–10 denticles and lower margin with 8–9 denticles (Fig. 22). Hind femur 3.5–3.7 times the pronotum length, without ventral spines. Body coloration as in males, wings light brown or green–yellowish, ovipositor green.

#### Bioacoustics.

Males stridulate at dusk and during the night, rarely during daytime. The tegmino–tegminal stridulation consists of groups of 5–18 syllables (mean±SD: 8.9±3.48, n=30 ♂♂). A group lasts for 1288–4761 ms and successive groups are separated by an interval of 157–326 ms. Groups have a repetition rate of about 10–25 per minute, depending on the number of syllables.

Two types of syllables may be observed in a group: “A” type and “B” type. Both syllable types are produced when the male closes its tegmina. The song pattern may be formulated as “A...AB–A...AB–A...AB–A...AB” and so on, where the unit “A...AB” forms a distinct group of syllables, “...” means a variable number of “A” syllables and “–” means the interval between successive groups of syllables. The “A” type syllable is formed of a compact series of 9–29 impulses (mean±SD: 18.6±6.31), lasting for 30–70 ms (mean±SD: 50.74±12.83). The “B” type syllable is formed of a compact series of 9–15 impulses (mean±SD: 12.36±2.1), lasting for 30–59 ms (mean±SD: 39.3±9.4) and always followed by a series of 1–4 after–clicks at an interval of 621–1655 ms (mean±SD: 1109.1±212.65). The production of the last syllable (“B” type) in each sequence is complex: the male partially closes tegmina, then holds them half–closed for about 621–1655 ms, and finally completely closes the wings with the after–clicks. This late production of after–clicks may function as the trigger element for female acoustic answer. Another possibility is that longer silent gaps may help the male to save up energy or simply listen to its environment, in order to detect possible threats or other singing males (Orci et al. 2010b, Szövényi et al. 2012).

The impulse interval is about 3–6 ms in all syllables and the acoustic signal slowly decreases in amplitude from beginning to end. In a group, the following syllable begins 60–131 ms (mean±SD: 96.28±24.62) later (Figs 23, 24). The carrier wave has the strongest components between 20–40 kHz, with the highest peak at about 29 kHz.

Females find males by phonotaxis and if willing to mate with the singing male (Fig. 7), they produce isolated impulses. In the resulting male–female duet, the female stridulates only after the male after–clicks (ending part of “B” syllable), with a latency of 13–34 ms (mean±SD: 25.66±6.42, n=12 responses from 3 females) (Fig. 25).

#### Distribution and ecology.

*Isophya dochia* sp. n. populates mesophytic subalpine meadows at about 1600–1900 m, in Ceahlău Mountain Massif, Eastern Carpathians (Fig. 11). The specimens were collected from leaves of *Urtica*, *Rubus*, *Veratrum*, *Rumex*, *Aconitum*, *Vaccinium*, *Hypericum*, *Stachys*, *Junniperus* etc. Few other bush–crickets and grasshoppers were found occurring simpatrically with the new species: *Metrioptera bicolor* (Philippi), *Metrioptera brachyptera* (Linnaeus),* Pholidoptera transsylvanica* (Fischer), *Miramella ebneri* (Galvagni), *Euthystira brachyptera* (Ocskay), *Myrmeleotettix maculatus* (Thunberg), *Chorthippus biguttulus* (Linnaeus), *Chorthippus parallelus* (Zetterstedt) etc. The bush–cricket *Isophya dochia* sp. n. has the same phenology as other subalpine *Isophya* species: female lays her eggs isolated in holes bitten in broad leaves of *Urtica*, *Rubus* etc. Eggs pass the winter in the litter and larvae hatch in late spring, after the snow melts in the high mountains. Depending on weather, first adults exuviate in late June and live up to August.

#### Etymology.

A noun in apposition; from the name of Dochia, a Romanian legendary female character based on an earlier deity of land and agriculture from the Dacian pantheon, and that of the eponymous rock in Ceahlău Mountains.

#### Discussions

(see Appendix 5: *Isophya harzi*, Appendix 6: *Isophya camptoxypha*, Appendix 7: *Isophya ciucasi*, Appendix 8: *Isophya sicula*, Appendix 9: *Isophya nagyi*).

Discovery of the bush–cricket *Isophya dochia* sp. n. is surprising, especially as it is a morphological cryptic species closely related to *Isophya camptoxypha*. Some other recently described species, *Isophya ciucasi*, *Isophya nagyi* and *Isophya sicula*, show high resemblance in morphology, while *Isophya harzi* Kis has a similar calling song (Figs 26–37). The song of these six species can be readily distinguished as syllables grouped in short sequences (*Isophya dochia* sp. n. and *Isophya harzi*) or arranged in series (*Isophya camptoxypha*, *Isophya ciucasi*, *Isophya nagyi* and *Isophya sicula*).

Both *Isophya dochia* sp. n. and *Isophya harzi* stridulate well defined assemblies of syllables (Figs 26, 27, 32, 33). Two other species have similar song patterns: *Isophya posthumoidalis* Bazyluk, distributed in Poland, Slovakia and N Romania (Szövényi and Orci 2008) and *Isophya beybienkoi* Mařan, known so far only from a very small area in SE Slovakia. In all these four species, males produce long sequences in which two types of syllables may be noticed: “A” and “B”, the rhythm of the whole song being a constant repetition that may be formulated as “A...AB–A...AB–A...AB” (in *Isophya dochia* sp. n. and*Isophya harzi*), “A...A–B–A...A–B–A...A” (in *Isophya posthumoidalis*) and “A...A–BA...A–BA...A” (in *Isophya beybienkoi*). The descriptions of calling songs of *Isophya posthumoidalis* and *Isophya beybienkoi* (Orci et al. 2001, Heller et al. 2004, Orci et al. 2010a) suggest that syllable types are very different from *Isophya dochia* sp. n.: in *Isophya posthumoidalis*, syllable “A” consists of a compact series of 5–10 impulses, lasting for 12–15 ms, and syllable “B” is a single impulse followed by 1–3 after–clicks, while in *Isophya beybienkoi* syllable “A” is formed of 5–13 impulses, lasting for 10-32 ms, and syllable “B” consists of a compact series of 2–9 short impulses followed by a longer one and 1–3 after–clicks. Not possessing any personal data on these two species, their songs and morphology were not illustrated in present paper.

In *Isophya camptoxypha*, the syllable is a short and compact series of impulses, lacking or followed by one (extremely rare 2–7) after–click (Figs 28, 34). Males of *Isophya ciucasi* stridulate a shorter syllable, but followed by a very high number of after–clicks, usually 10–30 (Figs 29, 35). *Isophya sicula* produces the shortest known syllables within this genus, consisting of only 1–3 impulses, missing or followed by one (extremely rare 2–5) after–click (Figs 30, 36). Finally, the song of *Isophya nagyi* is the most interesting, syllables being divided in two or three distinct fragments: the first one is an opening syllable and last two are part of the same closing syllable (Figs 31, 37). During our studies, less than 1% of analyzed syllables contained all three components, males usually producing sounds only when closing the tegmina.

Another interesting feature of the song in some of these species is the similarity of time–windows when after–clicks are produced: usually 50–80 ms after the syllable, but up to 151 ms in *Isophya camptoxypha* (Heller et al. 2004) and up to 110 ms in *Isophya sicula*. After–clicks may also follow both types of syllables of *Isophya harzi*. However, we observed a longer after–click delay (75–202 ms) next to the “B” syllable of the sequence and a shorter delay after the “A” syllables (40–90 ms). The longest after–click delay is found in *Isophya dochia* sp. n., up to 1655 ms.

A remarkable interspecific variation in this group is the number of teeth on male stridulatory file (Figs 63–68), which may be correlated with the total length and number of impulses in the unit syllable + gap + after–clicks, meaning a total closing stroke of wing. In the species that sing well defined groups of syllables: in *Isophya harzi*, 98–130 pegs produce a total number of 37–59 impulses lasting for 94–418 ms in the “B” syllable and 23–55 impulses lasting for 75–258 ms in the “A” syllable, while in *Isophya dochia* sp. n., the 82–89 pegs produce a total number of 9–29 impulses lasting for 30–70 ms in the first syllables from a group (“A” type) and 10–19 impulses lasting for 960–1770 ms in the last syllable (“B” type). In the species that stridulate ungrouped syllables: 65–85 pegs in *Isophya ciucasi* produce 13–56 impulses lasting for 209–438 ms, 50–80 pegs in *Isophya camptoxypha* produce 10–39 impulses, total duration of 27–363 ms, 48–60 pegs in *Isophya sicula* produce a total number of 1–8 impulses lasting for 52–265 ms. In *Isophya nagyi*, the high number of teeth on the stridulatory file, i.e. 102–109, produce a song of 36–108 impulses lasting for 167–793 ms (Table 2). All these differences in syllable production time are caused by slower or faster species–specific wing movements.

Female response as male acceptance is formed of isolated impulses, always produced after a particular part of male song, which supposedly acts as trigger. In both *Isophya dochia* sp. n. and *Isophya harzi* (n=13 responses from 3 ♀♀), the female replies only after the male’s “B” type syllable. The same behavior was noticed in females from *Isophya posthumoidalis* and *Isophya beybienkoi* (Orci et al. 2001, Orci et al. 2010a). Females of *Isophya camptoxypha*, *Isophya ciucasi* and *Isophya sicula* reply right after the male’s syllable main part. In *Isophya camptoxypha*, female response is produced immediately after the male’s after–click (n=19 responses from 3 ♀♀), while in *Isophya ciucasi* (n=37 responses from 6 ♀♀) and *Isophya sicula* (n=10 responses from 1 ♀) its answer is apparently not affected by male after–clicks. Females of *Isophya nagyi* stridulate only after the last component of the male syllable (n=25 responses from 4 ♀♀) (Figs 38–43).

Spectrographic analysis of sound reveals that in all six species the frequency ranges somewhere within interval 10–40 kHz, the maximum being recorded at about 20–30 kHz (Fig. 44).

In the six related species, males of *Isophya harzi* and *Isophya sicula* can be easily separated morphologically from *Isophya camptoxypha*, *Isophya ciucasi*, *Isophya nagyi* and *Isophya dochia* sp. n. *Isophya harzi* is more massive, males having shorter wings, marginal angle of tegmina less obtuse (110°), number of stridulatory teeth larger, and females having a longer ovipositor (11–13 mm). Males of *Isophya sicula* have a narrow left wing, similar with *Isophya posthumoidalis* and *Isophya beybienkoi* (Heller et al. 2004). In the other four species, the angle of cubital veins on male wing may be used as a differentiation tool: 80–90° in *Isophya ciucasi*, 70–80° in *Isophya nagyi*, 60–70° in *Isophya camptoxypha* and 70° in *Isophya dochia* sp. n.The tegmen marginal angle is almost constant in these species (about 120°), but less obtuse in *Isophya harzi* (110°) (Figs 45–50). In the six species, male cercus morphology has minute variations (Figs 51–56). The ovipositor is relatively similar in length in all species, being longer in *Isophya harzi* (Figs 57–62) and *Isophya beybienkoi* (Heller et al. 2004). Female stridulatory area is subject to minor intra– and interspecific variability (Figs 69–74).

Having a look at species distribution in the Romanian Carpathians, *Isophya camptoxypha* has the widest spread, inhabiting many Carpathian highlands. *Isophya harzi* is known only from two isolated mountains in the Southern Carpathians and most probably its distribution area is wider in the mountains between Prahova and Olt rivers. At this moment, both *Isophya ciucasi* and *Isophya dochia* sp. n. are known to have very restricted distribution areas: the isolated Ciucaş and, respectively, Ceahlău Mountain Massifs. Two species have been recently described from the Transylvanian volcanic mountains, in the Western part of the Eastern Carpathians. *Isophya nagyi* occurs in Călimani Mountains and Dorna Basin, while *Isophya sicula*, described from Harghita–Ciceu Mountains, has been recently found in the Moldavian Subcarpathians (Fig. 75). *Isophya posthumoidalis* was recorded in Romania only in Maramureş Basin (Szövényi and Orci 2008) and *Isophya beybienkoi* is known only from the area where it was described: Zadiělská planina and Plešivecká planina, Slovenskie Kras, SE Slovakia (Orci et al. 2001, Heller et al. 2004). In order to presume the evolutionary pattern within this group of species, some interesting areas must be taken into account, namely the regions where species live syntopically: *Isophya camptoxypha* and *Isophya nagyi* have been found together in W Călimani Mountains, *Isophya camptoxypha* and *Isophya sicula* in Moldavian Subcarpathians, *Isophya camptoxypha* and *Isophya ciucasi* in Central Ciucaş Mountains.

The Orthoptera species of the Carpathians have been well investigated and morphologically characterized. Yet, *Isophya*, and particularly *Isophya camptoxypha* and its allies, remain among the most intriguing and widely distributed bush–crickets in these mountains. Due to *Isophya camptoxypha*’s high intra– and interpopulational morphological variability, the main tool to separate correctly possible new cryptic species remains the oscillographic analysis of acoustic signals.

**Table 1.** Studied morphological characters (see Material and Methods) O – ovipositor.

		**HL (mm)**	**HW (mm)**	**PL (mm)**	**PW (mm)**	**TL (mm)**	**TW (mm)**	**BL (mm)**	**FL (mm)**	**CL (mm)**	**O (mm)**
male	Minimum	1.87	3.19	3.34	3.89	3.8	3.83	21.1	14.57	2.4	–
	Maximum	1.91	3.45	4.02	4.25	4.43	4.29	24.6	16.38	2.6	–
	Mean	1.89	3.32	3.8	4.07	4.23	4.07	22.32	15.2	2.51	–
	S. D.	0.02	0.1	0.24	0.14	0.23	0.16	1.12	0.61	0.08	–
female	Minimum	2.79	3.64	4.02	4.14	1.68	–	22.67	14.71	1.25	8.32
	Maximum	3.07	3.98	4.58	4.66	2.39	–	26.23	16.56	1.64	8.96
	Mean	2.9	3.8	4.31	4.37	1.92	–	24.44	15.36	1.42	8.61
	S. D.	0.11	0.11	0.18	0.21	0.25	–	1.31	0.66	0.15	0.21

**Table 2.** Examined male song characters in close related *Isophya* species (for abbreviations see Appendix 1: *Isophya* song abbreviations) n – number of analyzed specimens; t – temperature °C.

**Song type**	**Species**	**Song characters**	**Min.**	**Max.**	**Mean**	**S. D.**	**n**	**t**
Grouped syllable sequences	*Isophya dochia* sp. n.	ASD (ms)	30	70	50.74	12.83	30	20–25
		NIA	9	29	18.6	6.31		
		GS (ms)	60	131	96.28	24.62		
		BSD (ms)	30	59	39.3	9.4		
		NIB	9	15	12.36	2.1		
		DACB (ms)	621	1655	1109.1	212.65		
		NACB	1–4					
		BSTD (ms)	621	1770	1114.46	325.45		
		SGTD (ms)	1288	4761	2633.56	792.48		
		SGD (ms)	612	3028	1406.86	598.95		
		GSG (ms)	157	326	225.2	52.66		
	*Isophya harzi*	ASD (ms)	75	168	121.64	28.36	16	19–26
		NIA	23	55	40.5	9.67		
		DACA (ms)	40	90	64	16.97		
		NACA	0–2					
		ASTD (ms)	75	258	156.42	77.01		
		GAA (ms)	122	298	175.78	48.25		
		GAB (ms)	367	789	527.4	126.18		
		BSD (ms)	94	216	154.7	43.54		
		NIB	37	59	46.5	8.38		
		DACB (ms)	75	202	142.3	47.19		
		NACB	0–2					
		BSTD (ms)	94	418	223.3	114.78		
		SGTD (ms)	617	6168	2254.8	1954.04		
		SGD (ms)	617	6091	2126	2001.43		
		GSG (ms)	191	303	249.8	40.28		
Ungrouped syllable sequences	*Isophya camptoxypha*	SD (ms)	27	90	61.76	21.37	58	19–27
		NI	10	32	19.9	7.62		
		GS (ms)	110	289	189	50.18		
		DAC (ms)	56	257	112.6	70.71		
		NAC	0–1 (rarely 2–7)					
		STD (ms)	27	363	77.8	97.58		
	*Isophya ciucasi*	SD (ms)	7	24	15.56	5.12	34	24–28
		NI	3	26	15.32	6.74		
		GS (ms)	134	600	378.08	116.95		
		DAC (ms)	52	167	101.2	38.31		
		NAC	10–30					
		STD (ms)	209	438	273.76	56.76		
	*Isophya nagyi*	SD (ms)	167	793	486.28	200.49	12	21–25
		SDC1 (ms)	9	31	21	8.08		
		NIC1	5	18	12	4.72		
		GC12 (ms)	68	301	187.6	101.94		
		SDC2 (ms)	127	221	173.9	31.57		
		NIC2	35	79	57	13.5		
		GC23 (ms)	39	97	67.5	19.04		
		SDC3 (ms)	1	35	16.4	12.33		
		NIC3	1	11	5.1	3.36		
		GS (ms)	59	235	154.26	49.59		
	*Isophya sicula*	SD (ms)	1	4	1.6	1.03	3	25
		NI	1	3	1.3	0.65		
		GS (ms)	190	446	337.78	62.64		
		DAC (ms)	51	110	73.2	19.71		
		NAC	0–1 (rarely 2–5)					
		STD (ms)	52	265	75.1	63.96		

**Figures 6–11.**
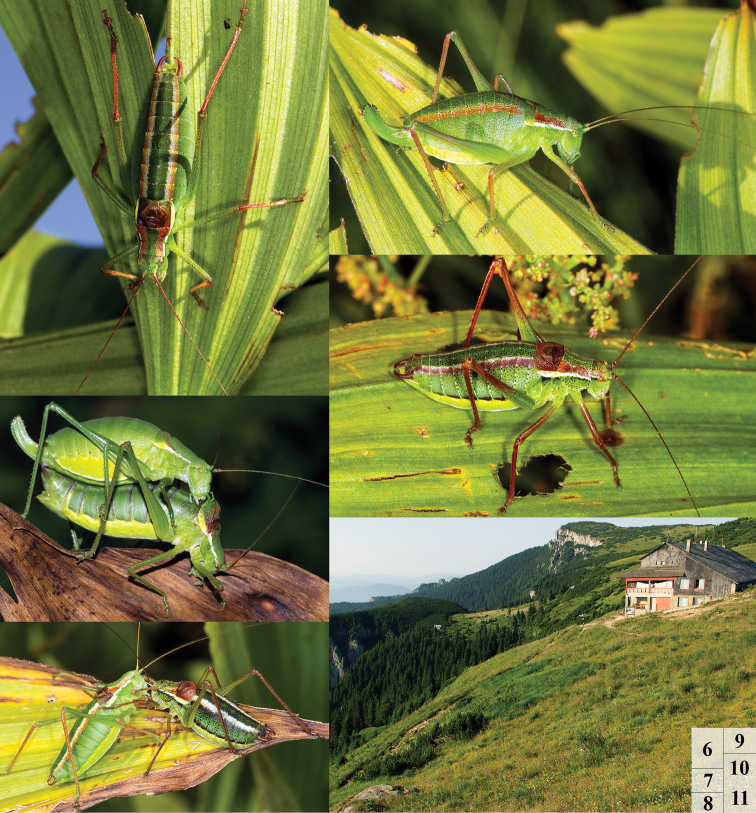
*Isophya dochia* sp. n.: **6** male habitus **7** copula **8** males’ rivalry **9** female habitus **10** male habitus **11** habitat in Ceahlău Mountains, near Dochia cabin (1740 m).

**Figures 12–22. F3:**
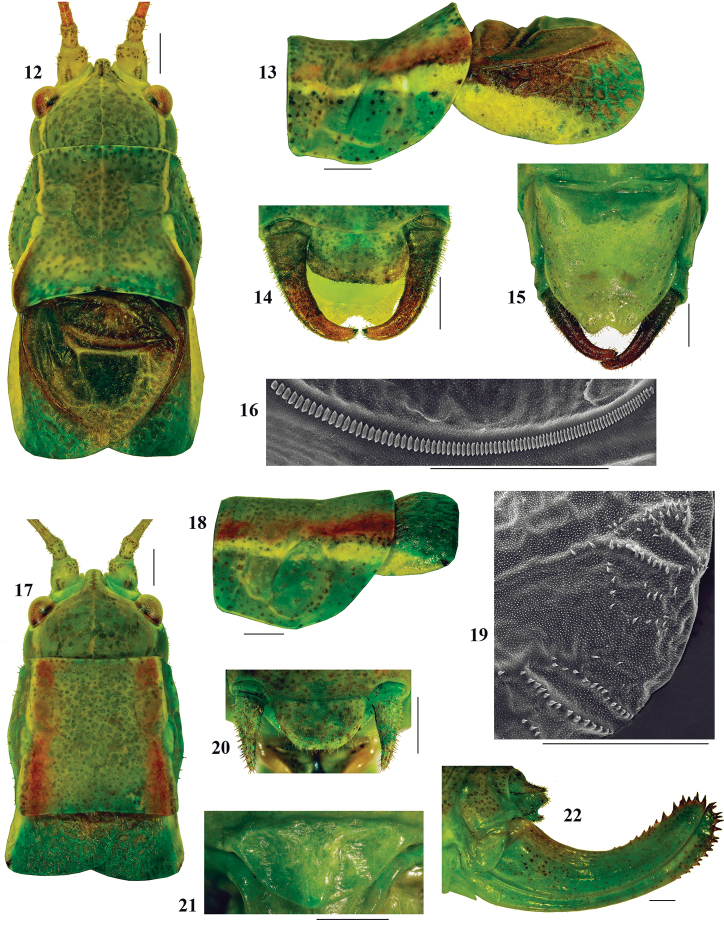
*Isophya dochia* sp. n.: **12** dorsal view of male head, pronotum and tegmina **13** lateral view of male pronotum and tegmina **14** male cerci **15** male subgenital plate **16** male stridulatory file (SEM photo) **17** dorsal view of female head, pronotum and tegmina **18** lateral view of female pronotum and tegmina **19** female stridulatory bristles (SEM photo) **20** female cerci **21** female subgenital plate **22** ovipositor. Scale 1 mm.

**Figures 23–25. F4:**
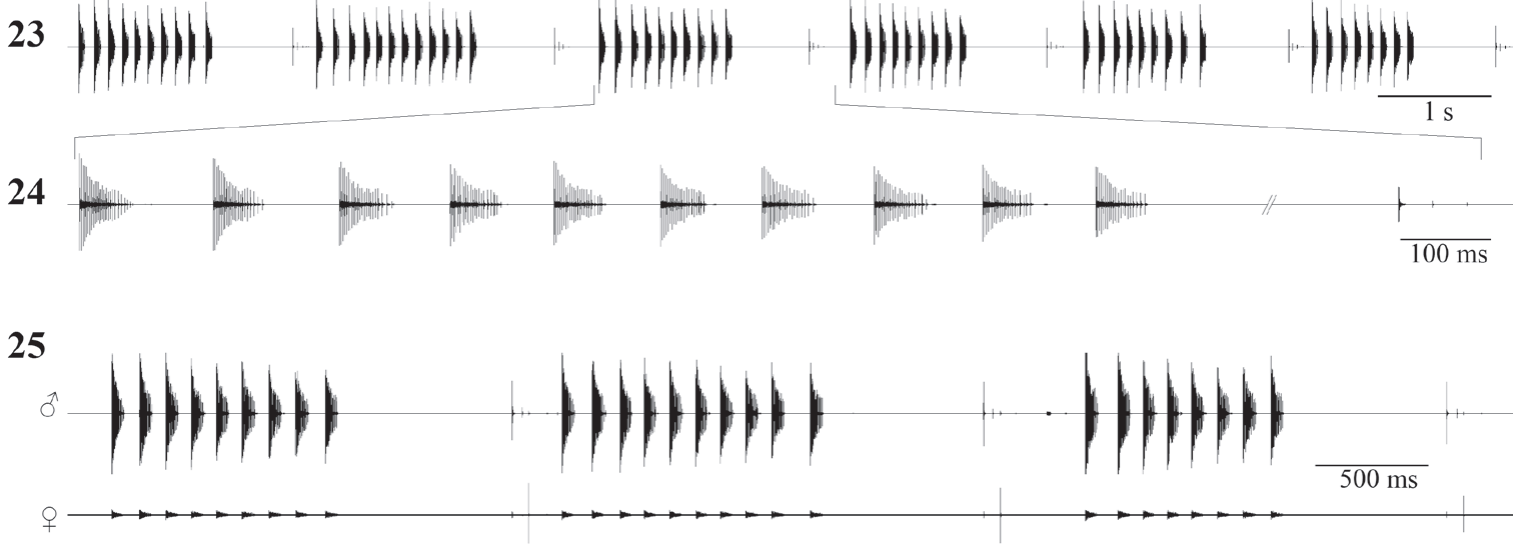
Oscillographic sound analysis in *Isophya dochia* sp. n., Ceahlău Mountains (24°C): **23** male song, consisting of syllable groups **24** detailed group of syllable **25** male–female mating acoustic duet.

**Figures 26–31.**
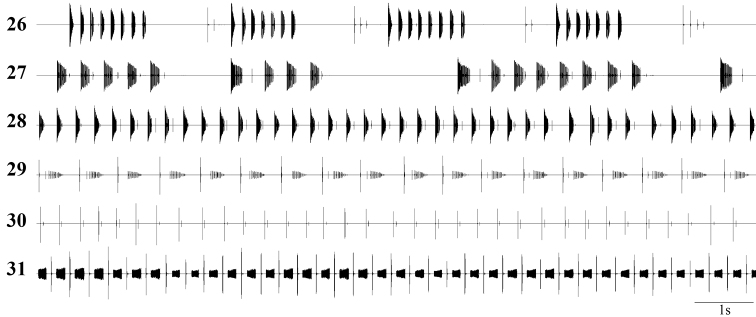
Oscillographic sound analysis: **26** *Isophya dochia* sp. n., Ceahlău Mountains (24°C) **27 ***Isophya harzi*, Cozia Mountains (25°C) **28** *Isophya camptoxypha*, Vânători Neamţ(26°C) **29** *Isophya ciucasi*, Ciucaş Mountains(26°C) **30** *Isophya sicula*, Harghita Mountains (25°C) **31** *Isophya nagyi*, Călimani Mountains (25°C).

**Figures 32–37.**
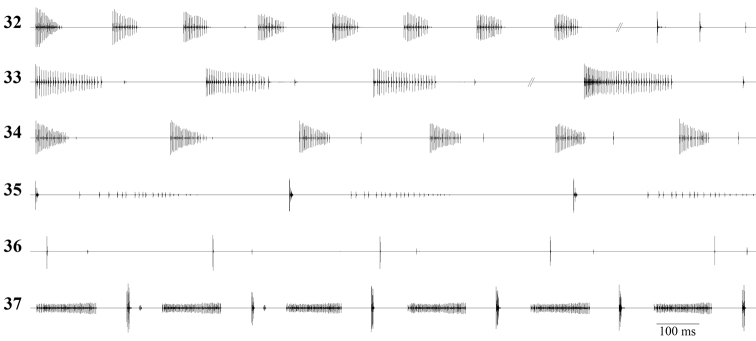
Detailed syllables: **32** *Isophya dochia* sp. n. **33** *Isophya harzi* **34** *Isophya camptoxypha* **35** *Isophya ciucasi* **36** *Isophya sicula* **37** *Isophya nagyi*.

**Figures 38–43.**
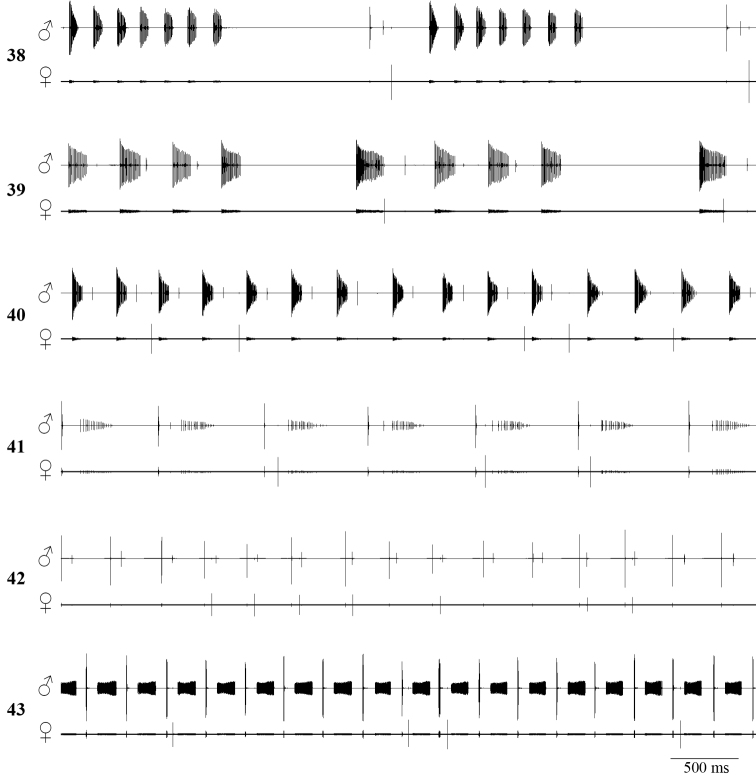
Male–female duet: **38** *Isophya dochia* sp. n., Ceahlău Mountains (24°C) **39** *Isophya harzi*, Cozia Mountains (25°C) **40** *Isophya camptoxypha*, Pleşa(24°C) **41** *Isophya ciucasi*, Ciucaş Mountains(24°C) **42** *Isophya sicula*, Harghita Mountains (25°C) **43** *Isophya nagyi*, Călimani Mountains (25°C).

**Figure 44.**
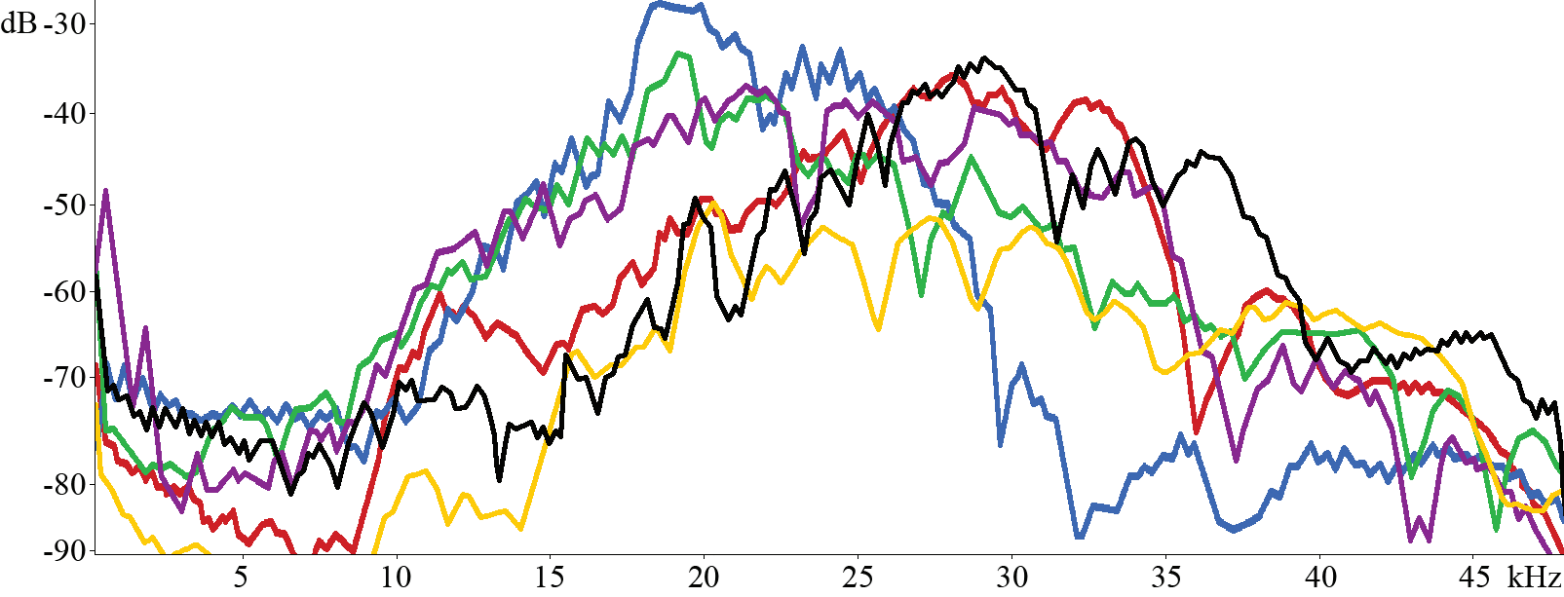
Sound spectrum: black **–** *Isophya dochia* sp. n.; blue **–** *Isophya harzi*; red **–** *Isophya camptoxypha*; green **–** *Isophya ciucasi*; yellow **–** *Isophya sicula*; purple **–** *Isophya nagyi*.

**Figures 45–74.**
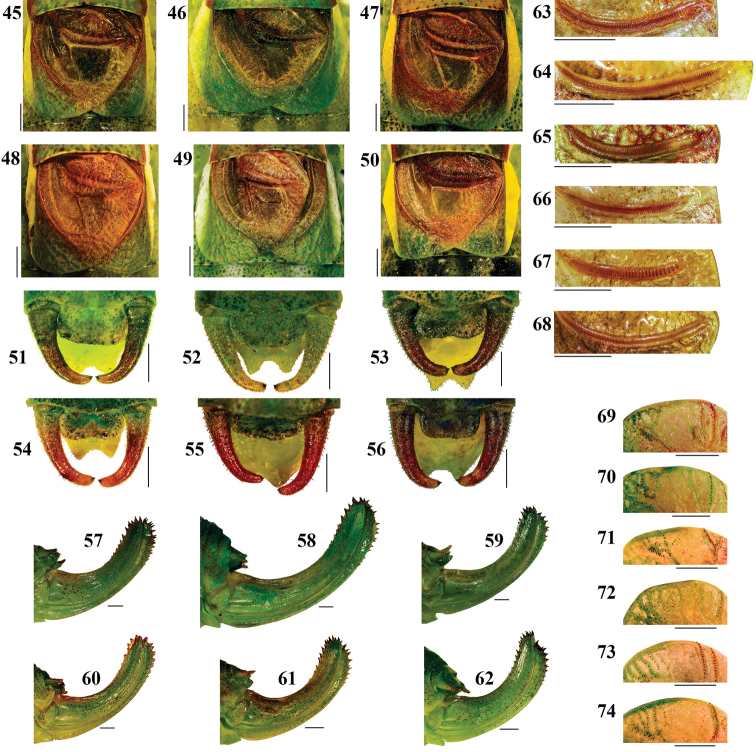
Morphological differences in close–related *Isophya* species: **45, 51, 57, 63, 69** *Isophya dochia* sp. n. **46, 52, 58, 64, 70** *Isophya harzi*, Piatra Craiului Mts. **47, 53, 59, 65, 71** *Isophya camptoxypha*, Nemira Mts. **48, 54, 60, 66, 72** *Isophya ciucasi*, Ciucaş Mts. **49, 55, 61, 67, 73** *Isophya sicula*, Harghita Mts. **50, 56, 62, 68, 74** *Isophya nagyi*, Călimani Mts. (**45–50** male tegmina **51–56** male cerci**57–62** ovipositor **63–68** male stridulatory file **69–74** female stridulatory bristles). Scale 1 mm.

**Figure 75.**
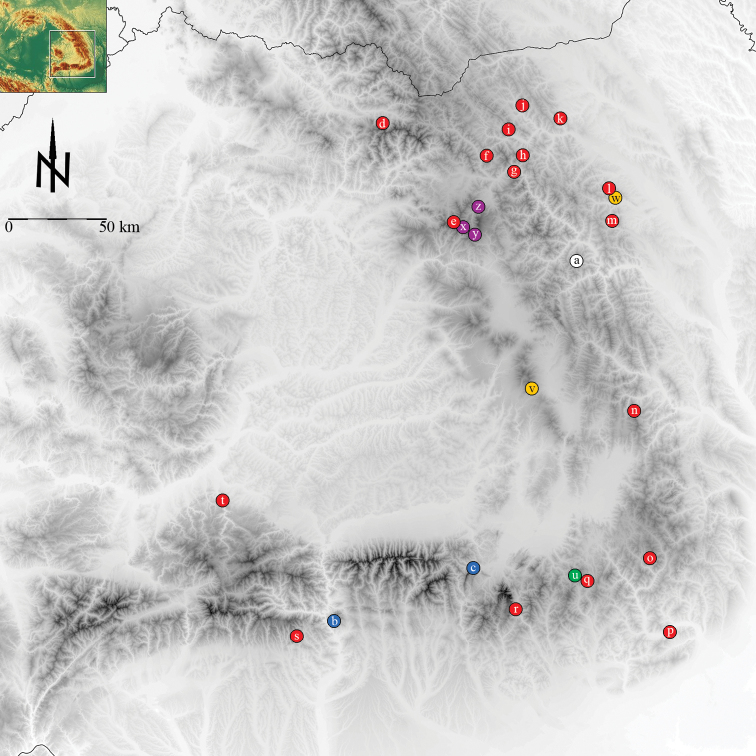
Distribution map of *Isophya camptoxypha* and allied species in the Romanian Carpathians, based only on acoustic analysis: white – *Isophya dochia* sp. n. (**a**); blue – *Isophya harzi* (**b, c**); red – *Isophya camptoxypha* (**d, e, f, g, h, i, j, k, l, m, n, o, p, q, r, s, t**); green – *Isophya ciucasi* (**u**); yellow – *Isophya sicula* (**v, w**); purple – *Isophya nagyi* (**x, y, z**). (**a** Ceahlău Mts. **b** Cozia Mts. **c** Piatra Craiului Mts. **d** Rodnei Mts. **e** Călimani Mts., Pietrosul peak **f** Mestecăniş **g** Pietrosul Bistriţei **h** Rarău **i** Sadova **j** Moldoviţa **k** Pleşa **l** Vânători Neamţ **m** Sihla **n** Nemira Mts. **o** Penteleu Mts. **p** Ciuta **q** Muntele Roşu, Ciucaş Mts. **r** Bucegi Mts. **s** Buila–Vânturariţa Mts. **t** Căpâlna **u** Ciucaş Mountains **v** Harghita–Ciceu Mts. **w** Vânători Neamţ **x** Călimani Mts., Pietrosul peak **y** Călimani Mts., Iezerul Călimanului peak **z** Neagra Şarului).

## Supplementary Material

XML Treatment for Isophya dochia

## Appendix 1

*Isophya* song abbreviations. (doi: 10.3897/zookeys.254.3892.app1) File format: Microsoft Excel file (xls).

**Explanation note:** Abbreviations used for calling songs of *Isophya* males, described in Figures 1-4 and Table 2.

## Appendix 2

*Isophya dochia* sp. n. audio. (doi: 10.3897/zookeys.254.3892.app2) File format: Windows audio file (wav).

**Explanation note:** Audio recording of calling song of a male *Isophya dochia* sp. n.

## Appendix 3

*Isophya dochia* sp. n. video. (doi: 10.3897/zookeys.254.3892.app3) File format: Video file (MPEG Layer 4) (mp4).

**Explanation note:** Video recording of calling song of a male *Isophya dochia* sp. n.

## Appendix 4

*Isophya* comparative material. (doi: 10.3897/zookeys.254.3892.app4) File format: Microsoft Excel file (xls).

**Explanation note:** Acoustic analyzed *Isophya* material in the Romanian Carpathian Mountains.

## Appendix 5

*Isophya harzi* audio. (doi: 10.3897/zookeys.254.3892.app5) File format: Windows audio file (wav).

**Explanation note:** Audio recording of calling song of a male *Isophya harzi*.

## Appendix 6

*Isophya camptoxypha* audio. (doi: 10.3897/zookeys.254.3892.app6) File format: Windows audio file (wav).

**Explanation note:** Audio recording of calling song of a male *Isophya camptoxypha*.

## Appendix 7

*Isophya ciucasi* audio. (doi: 10.3897/zookeys.254.3892.app7) File format: Windows audio file (wav).

**Explanation note:** Audio recording of calling song of a male *Isophya ciucasi*.

## Appendix 8

*Isophya sicula* audio. (doi: 10.3897/zookeys.254.3892.app8) File format: Windows audio file (wav).

**Explanation note:** audio recording of calling song of a male *Isophya sicula*.

## Appendix 9

*Isophya nagyi* audio. (doi: 10.3897/zookeys.254.3892.app9) File format: Windows audio file (wav).

**Explanation note:** Audio recording of calling song of a male *Isophya nagyi*.
